# Evolutionary foundations for cancer biology

**DOI:** 10.1111/eva.12034

**Published:** 2013-01-21

**Authors:** C Athena Aktipis, Randolph M Nesse

**Affiliations:** 1 Center for Evolution and Cancer, University of CaliforniaSan Francisco, CA, USA; 2 Department of Psychology, Arizona State UniversityTempe, AZ, USA; 3 Department of Psychiatry, The University of MichiganAnn Arbor, MI, USA

**Keywords:** darwinian, ecological, evolutionary medicine, mismatch, neoplastic, vulnerability

## Abstract

New applications of evolutionary biology are transforming our understanding of cancer. The articles in this special issue provide many specific examples, such as microorganisms inducing cancers, the significance of within-tumor heterogeneity, and the possibility that lower dose chemotherapy may sometimes promote longer survival. Underlying these specific advances is a large-scale transformation, as cancer research incorporates evolutionary methods into its toolkit, and asks new evolutionary questions about why we are vulnerable to cancer. Evolution explains why cancer exists at all, how neoplasms grow, why cancer is remarkably rare, and why it occurs despite powerful cancer suppression mechanisms. Cancer exists because of somatic selection; mutations in somatic cells result in some dividing faster than others, in some cases generating neoplasms. Neoplasms grow, or do not, in complex cellular ecosystems. Cancer is relatively rare because of natural selection; our genomes were derived disproportionally from individuals with effective mechanisms for suppressing cancer. Cancer occurs nonetheless for the same six evolutionary reasons that explain why we remain vulnerable to other diseases. These four principles—cancers evolve by somatic selection, neoplasms grow in complex ecosystems, natural selection has shaped powerful cancer defenses, and the limitations of those defenses have evolutionary explanations—provide a foundation for understanding, preventing, and treating cancer.

## Introduction

Our understanding of cancer is in the midst of a major transition. Extraordinary recent progress in genetics and cell biology is revealing details about cancer that undermine prior conceptions, and highlight the value of an evolutionary perspective. The naïve notion that cancer is one entity, with one cause, for which we can find a single cure, is fading as the complex and dynamic nature of cancer is becoming better understood (Gatenby 2009; Greaves and Maley 2012). The emerging view recognizes cancers as heterogeneous collections of cells (Campbell et al. 2008; Park et al. 2010; Maley et al. 2006; Merlo and Maley 2010) that evolve in tumor microenvironments with complex ecologies (Bissell and Radisky 2001). A full understanding requires evolutionary and ecological theory and methods.

The utility of evolutionary medicine (Nesse and Stearns 2008; Gluckman et al, 2009) for understanding cancer is illustrated by four principles. The most obvious is that neoplasms are heterogeneous populations of cells that evolve via somatic evolution. This principle, and associated phylogenetic methods, is proving crucial to understanding tumor heterogeneity, and its significance for optimizing chemotherapy. A second principle is that the fitness of cells, like individuals, depends not only on their genotypes and phenotypes, it also depends on their interactions within complex ecosystems. Ecological theory is proving important for understanding factors that stimulate and suppress the growth of neoplastic cells. The third principle is that powerful defenses against cancer were shaped by natural selection starting about one billion years ago. Finally, evolutionary medicine (Nesse and Williams 1994) explains how the limits of these mechanisms arise from the trade-offs between the risks of cancer and the benefits of retaining dynamic tissue capacities for development and repair.

These four principles provide an evolutionary framework for understanding the origins and progression of cancer that is parallel to what the Hallmarks of Cancer framework (Hanahan and Weinberg 2000) provides for understanding cellular mechanisms involved in cancer. These perspectives are entirely complementary; we need to know not only how cell regulation mechanisms work but also how they evolved to be the way they are, and why they are not better able to protect us from disease.

Evolution explains how cancers arise from the differential survival and proliferation of mutant cells that promote their own replication at the expense of the rest of the body (Greaves and Kinlan 2000; Greaves and Maley 2012; Merlo et al. 2006; Pepper et al. 2009). A view of cancers merely growing is being replaced by recognition that they evolve according to well-understood principles of somatic selection, along trajectories that can be described by established methods for tracing phylogenies. This has practical applications for understanding the significance of heterogeneity within tumors, and implications for diagnosis and treatment.

Ecological theory is equally useful for understanding cancer progression and resistance to chemotherapy. The growth, suppression, and death of neoplastic cells are explained not only by their genotypes and phenotypes but also by the microenvironments they inhabit. Such microenvironments impose powerful selection forces on neoplastic cells, and those cells, in turn, induce changes in microenvironments. So, too, do chemotherapy treatments. No amount of mechanistic detail is sufficient to explain these interactions; ecological theory is crucial.

**Figure 1:**
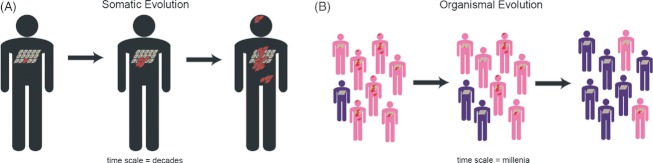
Evolution explains why cancer exists and also why it is not more common. (A) Cancer results from somatic selection at the cell level that favors neoplastic cells (red) over normal somatic cells (tan). (B) Cancer suppression results from selection at the organism level, favoring organisms that have traits (e.g., DNA repair, cell cycle checkpoints/apoptosis, and certain tissue architectures), which keep neoplastic cells in check (blue) while those individuals with traits that make them susceptible (pink) decrease in prevalence in the population.

Natural selection shaped mechanisms that suppress cancer remarkably effectively. With about 60 trillion cells in the human body, 500 billion of which are replaced each day (Cooper and Hausman 2009), it is amazing we do not all get cancer early in life. The relative rarity of cancer is even more remarkable when you consider the diversity of cells within many tumors, and the inevitability of somatic selection increasing the prevalence of the most malignant cells (Campbell et al. 2008; Park et al. 2010; Maley et al. 2006; Merlo and Maley 2010). The explanation goes back to the most important ‘major transition’ in the history of life—the origin of multicellular organisms about 1 billion years ago (Maynard Smith and Szathmáry 1995a). The tension between cell-level selection (somatic evolution) favoring neoplastic cells, and organism-level selection (organismal evolution) favoring individuals who are able to suppress mutations and rouge cells, was central. As organisms became longer lived, and the number of cells in a body increased from hundreds to trillions, suppressing cancer became ever more crucial (Caulin and Maley 2011; Nunney this issue). In short, evolution at the organism level shaped powerful mechanisms that suppress evolution at the somatic level (see Fig. 1).

Why are not those mechanisms better? The six kinds of evolutionary explanations for vulnerability to diseases in general all apply to cancer (Nesse 2005; Williams and Nesse, 1991). Evolutionary medicine attempts to understand the reasons why the systems of the body are limited in their capacities to protect us from disease. Nowhere is this better illustrated than in the diverse reasons why we remain vulnerable to cancer (Greaves 2007). As described in greater depth in the final section of this paper (see Box 1), cancer vulnerability can be understood within the larger framework of the six types of evolutionary explanations for traits that leave organisms vulnerable to disease:

1. Mismatch with novel environments (e.g., tobacco availability → lung cancer)
2. Co-evolution with fast-evolving pathogens (e.g.,. HPV → cervical cancer)
3. Constraints on what selection can do (e.g.,. mutations → cancer)
4. Trade-offs (e.g.,. capacity for tissue repair versus risk of cancer)
5. Reproductive success (RS) at the expense of health (e.g., cancer promoting alleles that may increase RS)
6. Defenses with costs as well as benefits (e.g.,. inflammation).

These four principles—somatic evolution of neoplasms, ecological analysis of tumor environments, selection for mechanisms that suppress cancer, and evolutionary explanations for their limits—provide an evolutionary foundation for understanding, preventing, and treating cancer. Research making use of these principles is well underway, but still at an early stage. The sections below illustrate the opportunities and some directions forward.

## Neoplasms evolve by somatic selection

### Cancer cells are heterogeneous

**Figure 2:**
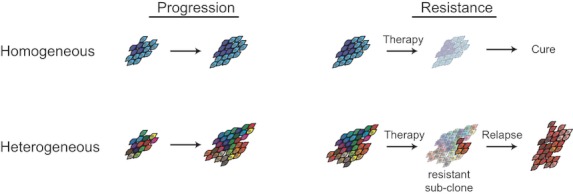
Intra-tumor heterogeneity increases the likelihood that some mutants will have a proliferation or survival advantage, resulting in faster progression. Heterogeneity also increases the likelihood that there will be a resistant mutant already present in the cell populations before therapy, making therapeutic resistance and relapse more likely.

Cancer is far from a single, well-defined disease. It is highly diverse, in ways more subtle than the obvious differences between cancers originating from different organs or tissues. For example, subtypes of breast cancer each have different risk factors, different phenotypic and genotypic characteristics, different effective treatment regimes, and different recurrence risks (Althuis et al. 2004; Bauer et al. 2007; Beaber et al. 2008; Colditz et al. 2004; Dawood 2010; Dolle et al. 2009; Foulkes et al. 2010; Koboldt et al. 2012). New genetic evidence confirms that cancers are highly diverse, even among cells within a single tumor. Genomics and single cell analyses in a variety of cancers show dramatic heterogeneity among cells (Campbell et al. 2008; Park et al. 2010; Maley et al. 2006; Merlo and Maley 2010; Anderson et al. 2011; Navin et al. 2011; Gerlinger et al. 2012; Nik-Zainal et al. 2012). Some types of cancers are characterized reliably by mutations in similar pathways or identical genetic alterations, such as the BCR-ABL translocation (Melo and Barnes 2007) or RB mutation in retinoblastoma (Dyer and Bremner 2005), but even these cancers can be highly diverse with regard to other mutations. Intra-tumor heterogeneity is critical because it is the raw material upon which somatic selection can act. Understanding cancers as intrinsically diverse is crucial because of the importance of heterogeneity in cancer progression and therapeutic resistance (see Fig. 2) (Campbell et al. 2008; Park et al. 2010; Maley et al. 2006; Merlo and Maley 2010).

### Origins of heterogeneity

While much attention is focused on heterogeneity that originated within neoplasms, theoretical calculations suggest that many mutations important for cancer may occur early in development, because a single early mutation may be transmitted to thousands or millions of daughter cells in a growing body (Frank 2010). This suggests that measures of heterogeneity may predict vulnerability to cancer, the need for close attention to the role of development in heterogeneity, and the importance of factors that influence the fidelity of replication during early developmental stages (Frank 2010; Meza et al. 2005).

Attention to development is equally important later in life. For instance, rates of breast cancer rise sharply in midlife, but more slowly later (American Cancer Society 2012), a pattern congruent with hormone induced cellular replication. However, similar patterns are found for other cancers, leading to the challenge of differentiating several possible explanations, including genetic heterogeneity (Frank 2007a).

### Heterogeneity and cancer progression

Within-tumor heterogeneity has an important implication for how somatic evolution proceeds: the more genetic variation in the population of cells, the more likely it is that some variants will have proliferative or survival advantages. Tumor diversity should therefore lead to faster progression to cancer—which is indeed what is observed in Barrett's esophagus (Maley et al. 2006; Merlo et al. 2010). Furthermore, tumor diversity is associated with clinical variables and histopathological characteristics associated with aggressiveness in breast cancer (Park et al. 2010).

One of the primary causes of tumor heterogeneity is genetic instability. Cancer cells exhibit a wide range of genetic modifications, from point mutations to massive chromosomal aberrations (Stephens et al. 2011). This results in many non-functional mutant cells, but a few whose genetic changes enhance their fitness. In Graham et al. (this issue), a model of the evolution of genetic instability finds cancer cells readily evolving a mutator phenotype. The mutator phenotype is suppressed by the resulting mutational load only under extreme parameters: where deleterious mutations are common, the cost of deleterious mutations is prohibitively high, and the benefit for driver mutations is extremely low. This surprising evolutionary viability of the mutator phenotype echoes other findings (Beckman and Loeb 2005) and is alarming given the carcinogenic effects of genetic instability. A separate model found that selection for driver mutations is more necessary for tumor growth early in progression than during later stages (Reiter et al. this issue).

Tumor heterogeneity is not only genetic; variations in the environments that tumor cells inhabit may be equally important. As is described in the next section on ecology, cancer cells inhabit complex microenvironments that vary substantially even within a tumor. For instance, Alfarouk et al. (this issue) call attention to the differences in availability of resources for cells living close to blood vessels versus those living farther away. They propose that heterogeneity in vascular density and blood flow are critical factors promoting cell heterogeneity.

### Heterogeneity and therapeutic resistance

Just as spraying fields with pesticides leads to selection for resistant pests, chemotherapy selects for resistant cells. Chemotherapy rarely kills every malignant cell. The chemo-resistant cancer cells that survive are selected for, and the chemo-sensitive cells are selected against. Heterogeneity of a tumor is therefore a likely to be a critical negative prognostic factor for chemotherapy outcomes, especially considering the known role of genetic instability in therapeutic resistance (Lee and Swanton 2012). After chemotherapy, resistant cells are not only more prevalent, they also have new ecological spaces into which they can expand, with potentially disastrous implications for patients.

Foo et al. (this issue) model tumor rebound growth following therapy and find that tumor diversity predicts early relapse when mutation rates are high. This may reflect faster evolution in cell populations with more genetic variation, or it may reflect the degree of genetic instability in the neoplasm. Because genetic instability is one of the primary causes of heterogeneity, and because tumor heterogeneity may increase genetically unstable variants, disentangling the roles of heterogeneity and genetic instability in cancer progression remains a major challenge. When mutation rates are lower, early relapse is associated with differences in the fitness of the sensitive and resistant cells rather than the diversity of the cells. These findings make it clear that the evolutionary processes underlying therapeutic resistance and relapse are complex and cannot be described by any simple generalization.

As discouraging as the seeming inevitability of the evolution of resistance might appear, considering evolutionary dynamics is essential for finding strategies to reduce resistance. For example, there is some evidence that lower dose chemotherapy conditionally applied only when a tumor is growing (the ‘adaptive therapy’ algorithm) leads to longer survival than the traditional high dose chemotherapy in a study of mice injected with ovarian cancer cells (Gatenby et al. 2009). This may be because lower dose chemotherapy does not select for chemo-resistant cells as strongly as high dose chemotherapy does, and sensitive cells may have a fitness advantage in the absence of therapy, leading to the maintenance of tumor cells that are responsive to chemotherapy. Another potential contributor to the apparent success of adaptive therapy may be that maintaining some of the tumor decreases new ecological openings for resistant cells to repopulate. In essence, adaptive therapy may prevent rapidly dividing resistant cells from taking over the population through a process analogous to ‘competitive release’ in ecology (Williams 2010). Several studies on adaptive therapy are currently in progress. If these efforts succeed, the adaptive therapy strategy could offer substantial clinical benefits.

### Heterogeneity and homogeneity

Emphasis on the importance of tumor heterogeneity by no means diminishes the importance of the continuing search for factors common to most neoplasms. It is equally important and unsurprising that mutations influencing cell cycle checkpoints, angiogenesis, apoptosis, and telomere synthesis are common across many neoplasms. The importance of heterogeneity also does not diminish the benefits of looking for genetic signatures characteristic of tumors in specific tissues, or signatures that define subtypes of cancers within a tissue, such as has recently been accomplished for breast cancer (Koboldt et al. 2012).

However, the search for shared factors and signatures that define specific subtypes of cancer is already proceeding at full speed. It seems to us that this effort to identify the signature of specific types of cancer sometimes tends toward essentializing types of cancer (see also Aktipis et al., 2010), as if all cases in one category are expected to be the same. In some respects they often are, and the classification of a cancer can provide important guides to treatment. However, an evolutionary approach encourages viewing variation among individuals, tumors, and cells as intrinsic to the process that gives rise to cancer, and to life itself, instead of as a limitation of our classification systems.

### Implications of tumor heterogeneity

Somatic evolution can act only when cells have heritable differences that influence survival or replication. Fisher's fundamental theorem of natural selection states that the rate of increase in a population's fitness is directly proportional to its genetic variation in fitness (Fisher 1930). The same principle applies to neoplasms; the more genetic diversity, the faster it evolves via somatic selection. This means that tumor diversity should influence cancer progression, not just for esophageal cancer, where the link has already been established (Maley et al. 2006; Merlo et al. 2010), but for all cancers. Further, heterogeneity is likely to emerge as a critical marker for resistance to chemotherapy (Lee and Swanton 2012). Future clinicians might be able to customize treatment based on tumor heterogeneity. As compared with traditional high dose chemotherapy, the adaptive therapy algorithm may extend survival over traditional high dose therapy, especially when tumors are highly heterogeneous and therefore likely to already harbor resistance mutations. Other strategies will also emerge from deeper understanding of somatic selection of heterogeneous cells.

## Cancers evolve in ecological contexts

### Overview of cancer ecology

**Figure 3:**
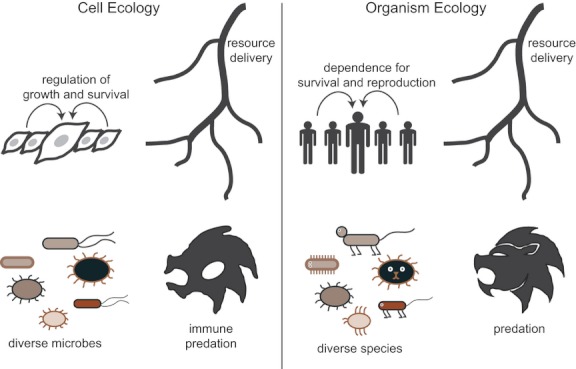
The ecological context of cancer cells parallels the ecological context for organisms. Similarities include dependence on limited resources, dependence on neighbors for survival and reproduction, interactions with other species and threats from predation. In the case of cancer cells, their ecological context is characterized by dependence resource delivery from blood vessels, growth and survival signals from neighbors, interactions with microbial species and predation from the immune system. Similarly, the ecological context for organisms is characterized by dependent on resource delivery from the environment, dependence on neighbors for effective survival and reproduction, interactions with other species and threats from predators.

The environments in which cancer cells live and evolve is as complex and multifaceted as the environments in which organisms evolve. The life of a cell in the body is characterized by complex development in a shifting ecosystem, followed by exposure to a variety of threats and opportunities, including attack by predatory immune cells, the limited availability of resources such as growth factors, oxygen, and glucose, and physical constraints and affordances shaped by adjacent cells and the basement membranes to which they are attached. Finally, many somatic cells live and evolve in environments teeming with diverse fast-evolving microbes. Taken together, these environmental and ‘social’ factors create a complex ecology that influences the fitness of somatic cells (Fig. 3) (Gatenby and Gillies 2008; Pienta et al. 2008). Indeed, the normal microenvironment of cells plays a critical role in cancer suppression, and changes to that microenvironment are a key factor in cancer initiation, progression and response to treatment (Correia and Bissell 2012; Nakasone et al. 2012; Pontiggia et al. 2012; Bissell and Hines 2011; Bissell and Radisky 2001).

The central roles of tumor microenvironment in suppressing and promoting cancer make ecological theory an essential tool for cancer researchers, as illustrated by four papers on the topic in this special issue (Thomas et al. this issue; Ewald and Swain Ewald this issue; Daoust et al. this issue; Alfarouk et al. this issue). Daoust et al. (this issue) describe applications of landscape ecology to cancer, offering methods for identifying tissue microhabitats that influence tissue growth, and vulnerability to metastasis. Similarly, Alfarouk et al. (this issue) describe how cells evolving near blood vessels (and their associated resources) are expected to evolve differently than those distant from blood vessels.

### Neighboring cells

The world of cancer cells is highly social. Tumor microenvironments include both other tumor cells as well as ‘normal’ cells nearby. These normal cells can be co-opted to provide growth signals or other fitness enhancing factors for the cancer cells. It remains known, for example, that the risk of cancer can be increased by a variety of changes to neighboring support cells or stroma (a combination of fibroblasts, vasculature, immune cells, and interstitial extracellular matrix) (Bissell and Hines 2011). Fellow cancer cells may also provide growth factors or engage in other actions that enhance their neighbor's fitness (Axelrod et al. 2006).

One paper in this issue (Sprouffske et al. this issue) argues that the maintenance of ‘non-stem cells’ in the tumor cell population may be explained by the positive influence of these cells on the fitness of genetically identical tumor-propagating cells (‘stem cells’). In other words, the model suggests that ‘non-stem cells’ could play a similar evolutionary role to ‘helpers at the nest’ in cooperative breeders (Alcock 2009), promoting their fitness indirectly through enhancing the fitness of their genetically identical parent ‘stem cell.’ The authors suggest that the promotion of tumor-propagating cells (‘stem cells’) by ‘non-stem cells’ may be similar to the promotion of the germ line by somatic cells in multicellular organisms. The importance of ‘stem cells’ or tumor-propagating cells for cancer progression is the focus of Greaves (Greaves this issue), who calls attention to the fact that stem cells are the unit of selection in cancer, an observation supported by the results of Sprouffske et al.'s (this issue) model.

The role that neighboring cells can have on each others' fitness also has implications for drug targeting. Drugs that disrupt the actions of secreted factors that benefit nearby cells (i.e., public goods) might slow development of therapeutic resistance to chemotherapeutic agents (Pepper 2012). Although cytotoxic drugs exert strong selection for cells that are resistant to therapy by killing as many cancer cells as possible, drugs that target public goods should not select strongly for resistant cells (Pepper 2012).

### Resource use and availability

Resource availability for a cell is influenced by both its own characteristics and by interactions with neighboring cells. For example, cancer cells that coordinate and regulate angiogenic signaling (for blood vessel growth) may induce greater blood flow to the tumor. Cells that do not coordinate angiogenic signaling may induce leaky vessels that temporarily increase blood flow (Nagy et al. 2012) until vessels collapse because of pressure loss resulting from excess permeability.

Some of these interactions among cells mimic characteristics of resource dilemmas in human populations, such as the Tragedy of the Commons (Hardin 1968), and can be profitably analyzed using game theory (Axelrod et al. 2006). Dilemmas such as these are characterized by conflict between individuals pursuing their own interests versus the interests of the group. Such dilemmas have important consequences for tumor evolution. For example, some models suggest that competition among cancer cells resulting in resource overuse may contribute to invasion and metastasis, just as high rates of resource use lead to dispersal of organisms (Aktipis et al. 2011). More generally, resource availability and distribution are expected to speed the evolution of cell motility in ways analogous to those observed for species (Chen et al. 2011). More subtle factors, such as the carrying capacity of the microhabitat, the quality of that habitat, and habitat fragmentation, also impose important selection forces that shape cancer cells (Daoust et al. this issue).

### Predation by immune cells

Organisms evolve capacities for evading their predators. Predators, in turn, evolve strategies for catching their prey despite all attempts at evasion. The resulting co-evolution shapes traits that can be understood only in light of their evolutionary histories. Somatic cells exposed to the threat of predation by immune cells vary in their ability to evade the immune system. Those that succeed best have a selective advantage, and they proliferate at the expense of others (Crespi and Summers 2005). This co-evolutionary process is ongoing in most cancers. Strategies malignant cells evolve for evading the immune system include mimicry, hiding, and co-opting immune cells in ways that speed cancer growth. (Gabrilovich and Pisarev 2003; Cavallo et al. 2011).

### Co-evolution with microbes

Cancer cells also co-evolve with cells from other species, namely the microbes that make up the microbiome. Human microbiome cells outnumber somatic cells 10 to 1 (Peterson et al. 2009). They are highly diverse, both among individuals and within a particular individual (Eckburg et al. 2005). Interactions with these microbes may, via somatic evolution, increase cancer vulnerability. The presence of certain microbes is associated with specific cancers including colorectal, gastric, oral/esophageal, mammary, lung, liver, and blood cancers (as reviewed by Arthur and Jobin 2011), suggesting that they may play important roles in cancer initiation and progression. The mechanisms underlying microbial influences on cancer are just starting to be understood, but it is clear that altered metabolism and immune system functions are important. It has long been known that inflammation increases susceptibility to cancer (Grivennikov et al. 2010), but recent work in mice suggests that bacterial colonization in distant tissues can alter gene expression elsewhere in the body, causing metabolic changes and inflammation in uninfected tissues (Rogers 2011). Microbes can also induce cells to switch from a stationary epithelial phenotype to a mobile mesenchymal phenotype called the Epithelial-Mesenchymal Transition (EMT) since microbes produce a variety of factors that can have effects on signaling pathways leading to EMT (Hofman and Vouret-Craviari 2012). This may have important implications for the transition from benign neoplasm to invasive and metastatic cancer.

### Exploitation by other species

Microbes can also influence neoplastic growth. One of the body's main defenses is mechanisms that kill infected cells, so it is not surprising that viruses can promote their own fitness through interfering with cell cycle arrest, apoptosis, telomere regulation, and cell adhesion (Ewald and Swain Ewald this issue). Such phenomena may explain how microbes can induce cancer. Cancer vulnerability should be increased by microbes that enhance their own fitness by increasing the proliferation of somatic cells that provide growth factors, immune protection, or physical niches in which the microbes thrive. In other words, while cancer may result from inflammation induced by microbes, it can also result from microbes increasing their own fitness by inducing somatic cell proliferation.

Crypts in the gastrointestinal tract provide niches for gut microbes, many of which are commensal and promote normal gut functioning (Yu et al. 2012). This raises the possibility that some cancers could result from side effects of mechanisms that microbes use to induce niche formation and expansion. Crypts produce mucus (Takubo et al. 1995; Levine et al. 1989) that promotes the survival of certain bacteria (Van den Abbeele et al. 2012; Hansson 2012), and colorectal adenomas are associated with mucosal adherent bacteria characterized by higher diversity (Shen et al. 2010), suggesting complex interactions between the cells that line crypts, and the bacteria that colonize the resulting niches.

This possibility might also be involved in Barrett's Esophagus, a premalignant condition characterized by the formation of crypts in the esophagus, where they are not normally present. During the process of neoplastic transformation, these crypts become longer and more tortuous (Srivastava et al. 2007). The formation of these crypts, and their subsequent lengthening, could result from microbes manipulating cell proliferation in ways that construct larger and more plentiful mucus-producing niches that benefit them, despite increasing cancer susceptibility for their host. Several findings support these speculative ideas, including (1) the association of esophageal cancer with specific microbes (Yang et al. 2009), (2) shorter crypts in germ free animals (Yu et al. 2012), and (3) longer crypts after oral inoculation with bacteria (Yu et al. 2012).

### Species extinction and cancer regression

Ecological theories describing species extinctions have implications for treating and preventing cancer. All individuals have neoplastic clusters of cells, most of which will never progress to cancer. Sometimes these growths spontaneously regress, a process analogous to species extinction. Tumor regression after treatment is also akin to extinction. Though treatment may cause regression, observations of spontaneous regression in the absence of treatment suggest that other mechanisms may also result in extinction of a malignant cell lineage. Applying ecological theories about species extinction to understanding why cancer cells regress (either spontaneously or as the result of treatment), suggests close attention to processes such as habitat destruction (Kareva 2011), competition, resource limitation, and factors that disrupt reproduction. Anti-angiogenic therapy limits blood supply to tumors, but has yielded mixed results, perhaps because the decrease in resource availability may select for dispersal or cell motility (Aktipis et al. 2012). Indeed, anti-angiogenic therapy may increase rates of metastasis (as reviewed in Grepin and Pages 2010). A more thorough ecological approach may help researchers anticipate these sorts of consequences and limit unintended negative effects.

### Implications of ecological theory for cancer research

Ecological theory offers tools and perspectives that help make sense of the complexities of cancer. Closer attention to the environment in which cancer evolves may suggest ways to slow cancer progression, enhance the effectiveness of treatment, or otherwise prolong life. These approaches include limiting the availability of resources for cancer cells, altering the social signals and context of neoplastic cells, and shaping interactions with microbes in ways that limit the fitness of neoplastic cells. They are parallel to approaches for understanding and limiting the abilities of pathogens to establish niches that allow them preferential access to resources (such as oxygen and glucose) and protection from threats (such as circulating immune cells). In the gastrointestinal tract “especially” perspectives from ecology, evolution, and microbiology intersect in ways with profound implications for understanding, preventing, and treating cancer.

## Organism-level evolution shapes cancer suppression

### Overview of cancer suppression

The story of cancer begins about one billion years ago at the dawn of multicellularity. Before the transition to metazoans, natural selection shaped one-celled organisms for whatever traits maximized their representation in future generations, especially maximal proliferation, invasion of adjacent spaces, and transmission to open niches. The transformation from unicellular to multicellular life was possible only when cells that cooperated by inhibiting their replication gained a selective advantage over those that went it alone (Maynard Smith and Szathmáry 1995b). How can cells that sacrifice their own capacity for replication succeed in competition with ‘selfish’ individual cells? Societies of cooperative cells can outcompete cells that try to go it alone. The success of these societies depends on their ability to suppress or kill cells that do not cooperate (Nunney this issue). This explains the evolution of the many mechanisms that suppress cancer, including effective DNA repair, cell cycle checkpoints, apoptosis, epigenetic modifications, and tissue architectures that limit the ability of over-proliferative cells to expand widely (Gatenby et al. 2010). Similarly, Ewald and Swain Ewald (this issue) argue that evolution has acted on multicellular organisms to create five primary barriers to the evolution of metastatic cancer: cell cycle arrest, apoptosis, limits to the number of cell divisions, cell adhesion, and asymmetric cell division. Cancer suppression mechanisms like these make it less likely that benign neoplasms will progress to cancer. In other words, evolution not only explains why cancer exists, it also explains why cancer is remarkably rare.

### The transition to multicellularity

Prior to the multicellular transition, mechanisms for inhibiting cell division were useless, with one crucial exception. In harsh environments, attempts to replicate are wasteful and may even kill a cell, so cells that can inhibit replication in these circumstances get a selective advantage. These mechanisms may have been co-opted in the transition to metazoan life, to regulate cell division during development, and to prevent cancer. This is consistent with genetic evidence for the continuity of mechanisms for programmed cell death from unicellular organisms to large multicellular organisms (Nedelcu 2009). During that transition, cancer suppression must have been a powerful selection force. Individuals with cells that divided out of control were at a severe selective disadvantage compared with those capable of controlling cell division and suppressing cancer to create functional multicellular bodies. The trade-offs between cancer suppression and functional multicellularity are central, not only to understanding cancer but understanding multicellular life itself.

### Peto's paradox and body size

Building complex multicellular organisms in the face of somatic evolution of cells in the body involves a multitude of trade-offs. As organisms became larger, longer lived, with orders of magnitude more cells, suppressing cancer became a larger problem. If we assume that every cell in a multicellular body has a certain chance of becoming malignant every year, then large, long-lived animals like elephants should have vastly higher rates of cancer than mice. However, this is not what we observe: large, long-lived animals seem to have similar (or lower) rates of cancer than small, shorter lived animals (Caulin and Maley 2011). This apparent inconsistency is known as Peto's paradox. What explains the unexpectedly low rate of cancer in large animals? Cancer itself has been a selection force that has shaped cancer suppression mechanisms that are as effective as they need to be, whatever the size of the organism. Indeed, emerging evidence suggests that, at least in the case of elephants, large animals may have more copies of tumor suppressor genes (Caulin and Maley 2011). Consistent with these findings, Roche et al. (this issue) describe a model showing that tumor suppressor genes are more likely to be activated more in animals with large body sizes. There are other possible explanations for Peto's paradox including the possibility that slower metabolic rates of larger organisms are protective (Caulin and Maley 2011). Investigations of Peto's paradox illustrate the power of the comparative method in evolutionary medicine.

Interestingly, if we look within species, size does influence cancer incidence. Tall humans are at significantly greater risk for cancer than shorter humans, with a 10 cm height increase leading to a relative risk of 1.1 for males and 1.14 for females. The same pattern holds for other species, such as dogs and rodents (as reviewed by Nunney this issue). This suggests that, within a species, large size should be correlated with cancer risk, but that among species cancer risks should be relatively similar because selection has shaped cancer suppression mechanisms including include DNA repair, immune surveillance, cell cycle checkpoint genes (such as p53), specialized tissue architecture, apoptosis, contact inhibition, and telomere length at the species level. As expected, small and large organisms differ in a variety of these mechanisms (as reviewed by Caulin and Maley 2011).

## Evolutionary explanations for cancer vulnerability

The story of the evolutionary origins of cancer and cancer suppression mechanisms is mostly one of selection and constraints. Cancer exists because cells were originally shaped to maximize replication. Cancer is rare because selection at the individual level has shaped powerful mechanisms to suppress cancer. The crucial remaining question is, why are not those mechanisms better? One major reason why cancer cannot be perfectly suppressed is that natural selection has constraints (see also Greaves 2007). Mutations happen, and path-dependence means that fundamental design limitations, such as the blind spot in the vertebrate eye, can never be corrected.

What about the other 5 evolutionary explanations for vulnerability to disease? (see Box 1) All of them contribute to explaining our vulnerability to cancer. Co-evolution with fast-evolving pathogens has already been discussed, with special emphasis on the benefits pathogens can get by inducing host cell replication. Closely related are defenses shaped by natural selection that contribute to cancer, especially the capacity for inflammation, with its unavoidable associated tissue damage. That leaves mismatch with modern environments and trade-offs, including the special trade-off of reproductive success at the expense of health.

**Text Box 1. Evolutionary Reasons for Disease Vulnerability Applied to Cancer**

Despite millions of years of evolution of cancer suppression mechanisms, we remain vulnerable to cancer. Nesse and Williams (Nesse 2005; Nesse and Williams 1994) offer six main reasons why natural selection leaves bodies vulnerable to disease (numbered below). Vulnerability to cancer can be understood within this same framework (bullets underneath numbers):

1. **Mismatch with the modern environment:** Selection is too slow to adapt bodies to rapidly changing environments, especially changes induced by human cultures.
  - Population migration and skin cancer (Jablonski and Chaplin 2010)
  - Caloric availability and obesity as a cancer risk factor (Wolin et al. 2010)
  - Higher availability of fats that promote tumor growth (Sauer et al. 2005)
  - Tobacco availability and smoking as a cancer risk factor (Peto et al. 2000)
  - Differences in number of reproductive cycles and breast cancer (Coe and Steadman 1995; Strassmann 1999; Eaton et al. 1994)
  - Exposure to light at night may increase breast cancer risk (Tomlinson et al. 2007; Blask et al. 2005).
2. **Co-evolution with pathogens:** Pathogens evolve much faster than larger organisms can, and co-evolution of pathogens and their hosts shapes extremely expensive and dangerous defenses.
  - The presence of specific microbes is associated with several specific cancers (as reviewed by Arthur and Jobin 2011)
  - Viruses induce some cancers (Ewald and Swain Ewald this issue)
  - Viruses integrated into our genome may influence cancer susceptibility (Tooby 2011)
3. **Constraints on selection:** Constraints on what natural selection can do are severe, including both limitations of space and time that apply to all systems, and the inability to start with a fresh design that limits organic but not mechanical systems.
  - Path-dependence in highly conserved cell cycle control mechanisms (Hartwell and Kastan 1994) may leave organisms susceptible to cancer or constrain therapeutic options
  - Constraints on the immune system's ability to detect cancer cells (Mapara and Sykes 2004) because cancer cells are derived from normal cells
4. **Trade-offs:** Changes that would make a trait less vulnerable to disease often lead to a decrease in fitness due to effects on other traits.
  - Fast and effective wound healing requires cell movement and proliferation (Guo and Dipietro 2010), capacities that leave an organism more vulnerable to cancer (Hofman and Vouret-Craviari 2012)
  - Fast growth may come at the cost of somatic maintenance, leading to cancer vulnerability (De Stavola et al. 2004)
5. **Reproduction at the cost of health:** Bodies are not shaped by natural selection for health or longevity; they are shaped to maximize reproductive success.
  - Competitiveness in males may lead to higher susceptibility to prostate cancer (Calistro Alvarado this issue)
  - Early menarche comes at the cost of higher susceptibility to breast cancer in females (Hsieh et al. 1990)
  - Women with BRCA mutations have greater susceptibility to breast cancer but also higher fertility (Smith et al. 2011)
6. **Evolved capacities for defense and their costs:** Many of the problems people bring to their physicians are not diseases themselves, but protective defenses shaped by natural selection such as cough, fever, pain, and vomiting. Like everything else, they have costs.
  - The capacity for inflammation is crucial not only for defending against infection but also for dealing with rouge cells. However, inflammation also damages tissues and makes them more vulnerable to cancer (Coussens and Werb 2002; de Visser et al. 2006).

### Mismatch

Cancer is not a disease exclusively of modern environments. Evidence for cancer in ancient mummies (Nerlich et al. 2006; David and Zimmerman 2010), to say nothing of other species, makes it obvious that cancer is not evolutionarily novel. Some kinds of cancer are, however, more common now than in ancestral times, often for obvious reasons. Lung and throat cancers increase dramatically in populations where smoking spreads, and decrease where smoking is curtailed (Peto et al. 2000). Mismatch between ancestral conditions and modern environments can also increase cancer rates when subpopulations move to environments different from those in which their ancestors evolved. Melanin pigmentation is a defense against skin damage, cancer, and perhaps degradation of folic acid as well. However, deeply pigmented skin also limits vitamin-D biosynthesis in environments with lower sun exposure. This is the leading explanation for the evolution of depigmented skin outside of the tropics (as reviewed by Jablonski 2004). The migration of light-skinned individuals to equatorial zones results in a mismatch between skin pigment and sun exposure that explains high rates of skin cancer.

More intriguing are increases in cancer rates arising in association with changes in reproductive patterns. Breast cancer rates are estimated to be more than ten times higher for women in the USA compared with hunter-gatherers (Eaton et al. 1994). This seems likely to result from increased hormone exposure, starting with earlier menarche, then augmented by contraception and years spent in reproductive cycles that would previously have been spent nursing babies. The average woman in the USA has over 400 menstrual cycles, compared with 100 in women in a pastoralist culture in Africa without birth control (Strassmann 1999). The role of hormone levels is supported further by a comparative study showing a high correlation between breast cancer rates and average levels of progesterone (Jasienska and Thune 2001).

When considering all cancers, the role of mismatch with modern environments is overwhelming. About one-third of cancers are direct complications of tobacco use, and another third are reported to be results of obesity, inactivity or poor diet (American Cancer Society 2012). In addition, many cancers are caused by radiation, hormone treatments, environmental exposures, and new pathogens. In contrast, only about 5% of cancers are products of hereditary genetic abnormalities. Cancer in modern populations is caused mainly not by the innate inadequacies of our bodies, but by exposure to aspects of modern environments for which our bodies are ill prepared.

Another important contributor to cancer rates in the modern environments is vastly extended average life span resulting from general good health and nutrition, and protection from infection. Hunter-gatherers who reach adulthood are very likely to live into their 60s or 70s, but prevention of early death has increased the average lifespan in modern societies by decades, and the proportion of people over 60 is many times larger than ever before. Cancer increases with age, as there are increased numbers of cell divisions, more accumulation of somatic mutations, and declining abilities to suppress cancer (Cancer Research UK 2012; American Cancer Society 2012). Some think that selection can have no effect after reproduction ceases, but this is incorrect; actions at any age that benefit kin who share your genes can influence an individual's contributions to the future gene pool. Nonetheless, the force of selection declines steeply starting at the age of first reproduction simply because some individuals die each year, even in the absence of aging and cancer. As a result, selection for mechanisms that suppress cancer fades to nearly nothing at advanced stages of life. As with cancer in general, the amazing thing is that the suppression mechanisms continue to work as well as they do at advanced ages.

### Trade-offs

Trade-offs are at the very center of evolutionary thinking. This is nowhere more evident than in applications to cancer. Organisms could have better mechanisms to prevent cancer, but the costs might be high for wound healing, growth, reproduction, and aging.

A key trade-off for any organism is between limiting uncontrolled cell division while maintaining the capacity to repair tissues. To heal a wound, cells must be able to proliferate and move (Guo and Dipietro 2010). However, having cells with the capacity to proliferate and move leaves an organism more vulnerable to cancer (Hofman and Vouret-Craviari 2012). Wound healing also requires the rapid generation of new blood vessels to nourish and oxygenate the healing cells (Guo and Dipietro 2010). Given that angiogenesis is one of the hallmarks of cancer (Hanahan and Weinberg 2000), the capacity for rapid angiogenesis is likely to increase vulnerability to cancer, despite its function in wound healing.

The trade-off between wound healing capacity and cancer suppression offers an important area for further work. In species that encounter greater physical threats (whether from high rates of injury from predators or high rates of intra-species aggression), one might expect that fast and effective wound healing to be a relatively stronger selective pressure than cancer suppression. Within a species, individuals with faster wound healing may be more vulnerable to cancer. Even within individuals, exposure to injuries or physical threats could conceivably shift physiological systems toward faster wound healing despite the increased risk of cancer. This hypothesis predicts that variations in the prevalence of injury among species (and perhaps among individuals within a species) may be associated with faster wound healing and higher cancer risk. These admittedly speculative suggestions can be tested using the comparative method, and they gain some support from evidence that aggression is associated with cancer risk in fish (Fernandez 2010), and that aggression may be associated with faster wound healing in baboons (Archie et al. 2012).

Embryogenesis and development are essential to multicellularity, but the cell capacities associated with these functions leave individuals vulnerable to cancer. Development involves ‘invasion’ of cells into other developing tissues during gastrulation. The capacity to transition from a stationary epithelial cell to a motile mesenchymal cell (a process known as the Epithelial-Mesenchymal Transition or EMT) is crucial to this process, but leaves organisms vulnerable to cancer (Hofman and Vouret-Craviari 2012).

Fast body growth and sexual maturation also increase cancer susceptibility. If replication is more accurate in slower growing organisms, they should have lower risks of cancer. The possibility that mutations early in development are especially important influences on cancer risk makes this doubly interesting (Frank 2007b). However, slower growth means…slower growth, thus delaying reproduction and reducing fitness. The advantages of faster growth may also have trade-offs resulting in increased risk of cancer because of less DNA repair, less apoptosis of cells with DNA damage, more generation of mutations earlier in development and perhaps higher levels of receptors for growth factors. This may explain why rates of breast cancer are higher for those with faster childhood growth (De Stavola et al. 2004), and early menarche (Hsieh et al. 1990), although increased hormone exposure may contribute as well.

Trade-offs between cancer and aging are illustrated by a tumor suppressor gene, p53 (TP53). Mice with supernumerary copies of p53 are protected from cancer, likely because they exhibit an enhanced response to DNA damage (Garcia-Cao et al. 2002). However, if the extra copies are constitutively expressed, mice show signs of premature aging (Tyner et al. 2002). If p53 is placed under proper regulatory control by its endogenous promoters, these super p53 mice do not age prematurely (Garcia-Cao et al. 2002), suggesting that aging and cancer are fitness trade-offs that have shaped the mechanisms that activate p53.

### Reproductive success at the expense of health

It is disturbing to recognize that natural selection does not shape organisms directly for health or longevity. An allele that increases net reproductive success will tend to increase in frequency irrespective of its effect on health. This phenomenon is most evident in the higher mortality rates in men compared with women in most modern societies. Success in mating competition has greater reproductive payoffs for males in many species, so natural selection has shaped investment in competitive abilities that are proportionately greater than investment in tissue repair capacities, as compared with women (Kruger and Nesse 2006). In developed societies, this results in early adult mortality rates three times higher for men than women.

The role of testosterone is evident in the correlation between testosterone levels and risk of prostate cancer. In a sophisticated analysis examining various factors contributing to epidemiological differences among human subgroups and rates of prostate cancer, Alvarado (this issue) notes that high levels of nutrition in modern Western cultures increases both investment in mate competition and testosterone levels. Subpopulations where competition is especially physical and desperate have higher testosterone levels and increased mating success, but at the cost of increased rates of prostate cancer. This thesis receives support from comparative studies showing higher rates of prostate cancer in human polygamous societies compared with monogamous cultures living adjacent in similar circumstances in Africa (Calistro Alvarado this issue). Trade-offs of this sort may exist for female susceptibility to breast cancer as well, as estrogen response to competitive interactions is associated with higher motivation for power (Stanton and Schultheiss 2007).

Many of the examples above fit neatly into evolutionary life history theory and its analysis of the trade-offs between somatic maintenance (i.e., acquiring resources and keeping one's body running in tip-top shape) and reproductive effort (i.e., acquiring mates, and making and caring for offspring) (Stearns 2000). In environments with high levels of extrinsic mortality, selection favors investment in early reproduction at the expense of somatic maintenance. Furthermore, environmental cues can calibrate these systems as a function of early experience or current conditions. Threats and uncertainty may shift investment from long-term reproductive goals to immediate survival goals, including up-regulation of the immune system and inflammation, more investment in wound healing, and speeding growth and reproductive maturity, even at the cost of higher long-term cancer risk. The life history trade-offs involved in differences in prostate cancer risk across populations noted by Alvarado (this issue) are a good example. For women, similar trade-offs may help to explain increased rates of breast cancer risk after exposure to stressful experiences, such as war exposure early in life (Keinan-Boker et al. 2009; Elias et al. 2004; Koupil et al. 2009). In mice, the stress of early social isolation leads to higher mammary cancer burden (Williams et al. 2009) and faster reproductive aging (Hermes and McClintock 2008).

### Implications of selection for cancer suppression, and its limits

Cancer suppression is as ancient as multicellular life. Multicellular organisms must have mechanisms to suppress cancer effectively. As evolution shaped larger and longer lived species, the problem of cancer suppression became more challenging, and the solutions for suppressing cancer became remarkably effective. They can never be perfect, however, for the same six evolutionary reasons that other bodily traits remain vulnerable to disease.

## Conclusion

The benefits of using evolutionary principles to understand cancer provide a specific example of the benefits of evolutionary medicine more generally. An evolutionary approach can help us understand why cancer exists and how it progresses (somatic evolution), how cancer cells interact with environments (ecological approaches), why it is not more common (natural selection for cancer suppression mechanisms), and why cancer suppression mechanisms can never be perfect (constraints, trade-offs, and other evolutionary reasons for vulnerability to disease). Evolution is essential for understanding cancer. It provides a framework for studying the evolutionary origins and progression of cancer that is parallel and complementary to the Hallmarks of Cancer framework for studying the mechanisms of cancer.

The importance of an evolutionary understanding cancer is not just an academic pursuit; it has great clinical utility that remains largely untapped. Evolutionary theory and methods have led to critical advances that promise to improve how we understand and treat cancer. For example, the finding that diversity in the premalignant biopsies predicts progression to cancer (Maley et al. 2006; Merlo et al. 2010) suggests methods for risk stratification, and a focus of clinical resources on those patients with the highest likelihood of cancer progression. Also, the development of novel therapeutic approaches, such as Gatenby's adaptive therapy algorithm (Gatenby et al. 2009), holds the promise of revolutionizing the way some cancers are treated—shifting the focus from eliminating every cancer cell, to controlling cancer by manipulating selection forces within the tumor. An evolutionary analysis of chemotherapy resistance suggests that taking another biopsy after a relapse may identify resistant mutations and guide targeted second line therapies. Finally, a clearer understanding of how large organisms suppress cancer (Caulin and Maley 2011), and the trade-offs inherent in cancer suppression, will inspire new strategies for risk assessment and cancer prevention. An example is provided by Hochberg et al.'s (this issue) discussion of new strategies to limit or eradicate incipient neoplasms by reducing microinflammation which may spur neoplastic progression, and by reducing the accumulation of DNA damage by administering poly ADP ribose polymerase inhibitors.

In retrospect, it is remarkable that the evolution of cells within tumors was not recognized until the 1970s with Nowell's (1976) paper ‘The clonal evolution of tumor cell populations.’ Despite subsequent wide acceptance of evolutionary explanations for cancer progression, applications of evolutionary thinking remain limited; for instance, evolutionary terms are used in only about 1% of the abstracts of papers on therapeutic resistance (Aktipis et al. 2011). While applications of evolutionary principles to the problems of cancer are in their infancy, they are growing fast, as illustrated by many recent conferences across the world, and the creation of two centers for the study of evolution and cancer, the Center for Evolution and Cancer at the University of California, San Francisco, and the Centre for Ecological and Evolutionary Cancer Research at University of Montpellier. We anticipate that evolutionary applications that advance cancer research and treatment will speed the growth of evolutionary medicine more generally, and that as more physicians have opportunities to learn the basic science of evolutionary biology, their insights will further advance our understanding of cancer, as well as the rest of medicine.
