# Corrigendum

**DOI:** 10.1111/eva.12063

**Published:** 2013-05-21

The article by Aktipis and Nesse (2013) contained an error where Leo Kinlen was wrongly attributed to Mel Greave's book, ‘Cancer: The Evolutionary Legacy’. The citation in the text should read ‘(Greaves 2000)’ rather than ‘(Greaves and Kinlen 2000)’.
